# SARS-related Perceptions in Hong Kong

**DOI:** 10.3201/eid1103.040675

**Published:** 2005-03

**Authors:** Joseph T.F. Lau, Xilin Yang, Ellie Pang, H.Y. Tsui, Eric Wong, Yun Kwok Wing

**Affiliations:** * The Chinese University of Hong Kong, Hong Kong Special Administrative Region, China

**Keywords:** Hong Kong, SARS, community responses, perceptions, mental health, Chinese

## Abstract

To understand different aspects of community responses related to severe acute respiratory syndrome (SARS), 2 population-based, random telephone surveys were conducted in June 2003 and January 2004 in Hong Kong. More than 70% of respondents would avoid visiting hospitals or mainland China to avoid contracting SARS. Most respondents believed that SARS could be transmitted through droplets, fomites, sewage, and animals. More than 90% believed that public health measures were efficacious means of prevention; 40.4% believed that SARS would resurge in Hong Kong; and ≈70% would then wear masks in public places. High percentages of respondents felt helpless, horrified, and apprehensive because of SARS. Approximately 16% showed signs of posttraumatic symptoms, and ≈40% perceived increased stress in family or work settings. The general public in Hong Kong has been very vigilant about SARS but needs to be more psychologically prepared to face a resurgence of the epidemic.

The severe acute respiratory syndrome (SARS) epidemic affected ≈30 countries, resulting in 8,422 cases and 916 deaths globally (1). Approximately 20.8% (1,755) of the cases and 32.8% (300) of the deaths occurred in Hong Kong. The World Health Organization issued a travel advisory warning against visiting Hong Kong from April 2 to May 23, 2003 (2). School classes were suspended from March to May 2003 (3). More than 90% of Hong Kong residents frequently wore face masks in public places from March through May 2003, and 33.6% worried that they or their family members would contract the disease (4). A number of hypotheses have been generated about different modes of transmission of SARS (5–7). However, responses to many of these issues have not yet been formulated. From December 16, 2003, through April 30, 2004, another 14 new SARS cases were reported in 4 areas in China (8–10). Public health measures played an important role in the control of the spread of SARS in the community (11,12). Whether SARS will reappear in some parts of the world is not known.

Studies of the psychological effect of disastrous events at a general population level have been reported. Some studies investigated the effect of the September 11, 2001, terrorist attack in the United States (13–16). Longitudinal studies found that some of the mental health problems could become chronic (15,17). Similar studies have been conducted for other disasters, such as the 1995 Sarin attack in the Tokyo subway system (18) and the terrorist attacks in Israel (19). SARS-related psychological problems have been reported to be prevalent in the general population (20,21).

The first objective of our study was to investigate how members of the general population in Hong Kong perceived different aspects of SARS and how people would react to a possible resurgence of SARS ≈6 months after the end of the epidemic (survey 1). The second objective was to assess the mental health effects on the general population at the end phase of the epidemic and to investigate relationships among various reactions, perceptions, and mental health effects (survey 2).

## Participants and Methods

The study population was composed of Chinese-speaking residents of Hong Kong (22) who were 18–60 years old. We conducted 2 independent cross-sectional telephone surveys to achieve the 2 aforementioned objectives. Survey 1 was conducted from December 30, 2003, to January 17, 2004, and survey 2 from May 27, 2003, to June 1, 2003. Telephone numbers were randomly selected from up-to-date residential phone directories. Nearly 100% of the Hong Kong residents have telephones at home (Hong Kong Office of the Telecommunications Authority, pers. commun.), and other local studies have used telephone surveys for data collection (21).

Interviewers called between 6:00 p.m. and 10:30 p.m. to avoid undersampling workers and students. If a telephone call was not answered, at least 2 follow-up calls were made at different hours on weekdays. An eligible household member, whose birthday was closest to the date of the interview, was invited to participate in the study. Verbal informed consent was obtained from participants. Ethical approval was obtained from the Chinese University of Hong Kong. The response rate, defined as the number of participants completing the survey divided by the number of valid households contacted, was 65% for survey 1 and 57.7% for survey 2. Relevant sociodemographic characteristics of respondents are shown in Table 1.

**Table 1:** Sociodemographic characteristics of respondents*

	Survey 1			Survey 2		
	Men (n = 428), %	Women (n = 435), %	Total (N = 863), %	Men (n = 407), %	Women (n = 411), %	Total (N = 818), %
Age group (y)						
18–29	25.6	22.4	24.0	35.5	24.1	29.8
30–44	37.2	44.8	41.0	33.0	50.5	41.8
45–60	37.2	32.8	35.0	31.5	25.4	28.4
Education level						
£9 y	23.7	32.5	28.1	24.0	32.0	28.0
10–12 y	48.4	44.9	46.6	44.4	47.3	45.9
Post secondary	27.9	22.6	25.2	31.6	20.7	26.1
Marital status						
Single	39.2	25.8	32.4	44.0	28.3	36.1
Married/divorced/widowed	60.8	74.2	67.6	56.0	71.7	63.9
Employment status						
Full time	71.1	42.3	56.6	65.4	41.4	53.3
Housewife/student	10.8	50.8	31.0	14.3	42.1	28.2
Other	18.1	6.9	12.5	20.4	16.5	18.5
Monthly income (HKD)						
£4,000	24.6	50.2	37.9	–	–	–
4,001–12,000	42.1	27.8	34.7	–	–	–
12,001–20,000	18.5	11.2	14.7	–	–	–
<20,001	14.9	10.8	12.7	–	–	–

*HKD, Hong Kong dollar (1 US$ = 7.8 HKD). –, data not collected in survey 2.

Respondents in survey 1 were asked about SARS-related perceptions, different public health measures currently practiced, and one's anticipated public health and emotional responses if SARS were to return to Hong Kong. Respondents in survey 2 were asked about psychological effects of SARS. These included whether respondents felt horrified, helpless, or apprehensive; had sleeping and psychosomatic problems; had increased smoking and alcohol consumption; or had perceived stress. The Chinese version of the Impact of Event Scale (IES) (23,24) and the mental health (5 items) and the vitality (4 items) subscales of the Medical Outcomes Study 36-Item Short Form Health Survey (SF-36) (25–27) were also used in survey 2.

In survey 1, multivariate logistic regression analysis, using univariately significant responses as input variables, was performed. In survey 2, 2-staged stepwise linear and logistic regression models were used. SPSS for Windows Release 11.0.1 (SPSS Inc., Chicago, IL, USA) was used and p values < 0.05 were considered significant.

## Results

### Current SARS-related Preventive Behavior

At the time of the first survey, 66.7% and 68.6% of the respondents, respectively, would avoid visiting hospitals or mainland China (Table 2). More than 80% would make a health declaration to customs, use a mask on a flight, or see a doctor when traveling overseas if they had influenza, while 38.7% would see a local doctor in mainland China under such circumstances (Table 2). Women were more likely than men to avoid visiting China or avoid seeing a local doctor if they had influenza when traveling overseas (p < 0.05) (Table 2).

**Table 2:** Perceptions related to resurgence of severe acute respiratory syndrome (SARS) and associated behaviors (survey 1 data)

Perceptions	Men (n = 428), %	Women (n = 435), %	Total (N = 863), %	p value*
Resurgence of SARS				
There will be a resurgence of SARS in Hong Kong in the coming 6 months	37.4	43.4	40.4	0.069
There will be a resurgence of SARS in China in the coming 6 months	66.6	71.3	68.9	0.138
There will be a resurgence of SARS overseas in the coming 6 months	29.9	28.3	29.1	0.613
No major outbreaks even if SARS returns to Hong Kong	71.3	68.3	69.8	0.340
The government could control SARS if there were a few sporadic new SARS cases in Hong Kong	80.3	80.2	80.3	0.958
Preventive behavior if a few new SARS cases were reported in Hong Kong				
Would frequently wear a mask in public places	70.7	71.7	71.2	0.730
Would avoid going to crowded places	71.5	77.9	74.8	0.031
Would avoid going to mainland China	69.6	79.7	74.7	0.001
Would not allow children to go to school	13.5	12.1	12.8	0.536
Would avoid going to hospitals	67.3	76.3	71.8	0.003
Would avoid contacts with medical personnel	35.8	38.4	37.1	0.437
Would avoid contacts with tourists coming from mainland China	31.5	37.8	34.7	0.051
Perceived emotional responses if a few new cases were reported in Hong Kong				
Would be in a state of panic	14.0	23.0	18.6	0.001
Would be very depressed	12.1	17.7	14.9	0.020
Am still emotionally disturbed because of SARS	33.0	39.5	36.3	0.047
Current preventive behavior				
Would avoid visiting hospitals to prevent contracting SARS	65.0	68.3	66.7	0.311
Would avoid visiting China to prevent contracting SARS	64.4	72.8	68.6	0.008
Would make a health declaration if crossing the border and had influenza	79.3	84.3	81.9	0.058
Would see a local physician if had influenza in mainland China	37.6	39.8	38.7	0.498
Would see a local physician if had influenza overseas	79.3	84.8	82.1	0.039
Would wear a mask if I had influenza when traveling by air	87.3	91.0	89.2	0.079

*Chi-square test.

### SARS-related Perceptions

From 65.0% to 89.3% of respondents believed that SARS could be transmitted through droplets, fomites, and sewage systems; by eating wild animal meat; or by rats, cockroaches, or pets, while 49.2% of respondents believed that SARS is transmittable through aerosols (Table 3). Of all respondents, >90% believed that using a mask in public places, disinfecting living quarters, and frequent hand washing are efficacious means of SARS prevention (Table 3).

**Table 3:** Perceptions related to mode of transmission, medical development, and epidemiology of severe acute respiratory syndrome (SARS) (survey 1 data)

% agreeing with statements	Men (n = 428), %	Women (n = 435), %	Total (N = 863), %	p value*
Mode of transmission				
SARS is transmittable through respiratory droplets	86.7	88.5	87.6	0.416
SARS is transmittable through fomites	87.1	87.6	87.4	0.847
SARS is transmittable through aerosols	47.2	51.3	49.2	0.232
SARS is transmittable through rats and cockroaches	70.6	79.3	75.0	0.003
SARS is transmittable through pets	66.6	63.4	65.0	0.333
SARS is transmittable through sewage	86.7	92.0	89.3	0.012
There is a high likelihood of contracting SARS through wild animal meat	77.4	87.6	82.6	<0.001
Perceived efficacy of preventive measures				
Wearing masks in public places could effectively prevent SARS	92.0	93.3	92.7	0.471
Disinfecting living quarters could effectively prevent SARS	96.0	98.6	97.3	0.018
Frequent hand washing could effectively prevent SARS	96.3	99.3	97.8	0.002
Vaccination against influenza could effectively prevent SARS	44.6	47.7	46.2	0.363
Intake of traditional Chinese medicine could effectively prevent SARS	36.6	44.2	40.5	0.023
Medical development				
SARS vaccines would be developed in a year	47.2	44.7	45.9	0.462
No effective drugs available to treat SARS	82.9	80.5	81.7	0.345
Epidemiology of SARS				
Old people are more likely than others to contract SARS	68.9	70.5	69.7	0.613
SARS mortality rate >50% for patients >60 years old	44.7	47.6	46.2	0.400

*Chi-square test.

A total of 40.4%, 68.9%, and 29.1% of the respondents, respectively, believed that resurgence of SARS would occur in Hong Kong, in mainland China, or overseas in the coming 6 months. In addition, 69.8% of respondents believed that even if this resurgence occurred, it would not be a major outbreak, and 80.3% believed that the government would be able to control the epidemic under such circumstances (Table 2).

In the event that a few new cases of SARS were reported in Hong Kong, >70% of all the respondents would wear a mask in public places and avoid visiting crowded places, mainland China, or hospitals (Table 2); 12.8% of respondents would not allow their children to attend school. A total of 37.1% of respondents would avoid contacting medical personnel, and 34.7% would avoid contacting visitors from mainland China. Furthermore, 18.6% of the respondents indicated that they would be in a state of panic, and 14.9% would be very depressed. Approximately 36.3% of the respondents felt emotionally disturbed because of SARS.

Female respondents were more likely than male respondents to perceive SARS to be transmittable through different modes (rats and cockroaches, animal meat, and sewage) or to perceive efficacy in disinfecting living quarters, washing hands frequently, and using traditional Chinese medicine for SARS prevention (p < 0.05) (Table 3). Women were also more likely than men to be in a state of panic and be depressed or emotionally disturbed because of SARS (p < 0.05) (Table 3).

### Factors Predicting Public Health Measures for Preventing SARS

Multivariate results show that sex, marital status, believing that SARS would be transmitted through fomites or aerosols, perceiving that older people were more susceptible to SARS, perceiving that a resurgence would occur in Hong Kong or in China, and current emotional disturbance because of SARS were associated with visiting hospitals or visiting mainland China ( Table A1). Sex; education level; marital status; believing that SARS was transmitted through droplets, fomites, pets, or sewage; anticipation of a resurgence in SARS in Hong Kong or overseas; and the perceived ability of the government to control the resurgence of SARS were associated with being emotionally disturbed by SARS or in a state of panic if SARS returned to Hong Kong (Table A1).

### Mental Health Effects of SARS

A total of 92.5% of the respondents regarded the SARS epidemic in Hong Kong as severe or very severe. High percentages (65.4%, 55.5%, and 65.0%, respectively) of respondents felt helpless, horrified, and apprehensive because of SARS or worried that they or family members would contract the virus, and 48.4% of respondents perceived that their mental health had severely or moderately deteriorated because of the SARS epidemic (Table 4).

**Table 4:** Psychological and related effects of severe acute respiratory syndrome (SARS) (survey 2 data)*

	Men, %	Women, %	Total, %	p value†
General mental health effect of SARS				
SARS perceived to be severe or very severe	91.4	93.7	92.5	0.216
Felt horrified because of SARS (agree or strongly agree)	65.4	80.3	72.9	<0.001
Felt apprehensive because of SARS (agree or strongly agree)	55.5	69.1	37.7	<0.001
Felt helpless about SARS (agree or strongly agree)	65.0	63.7	64.4	0.703
Worried or worried very much about oneself or family members contracting SARS	41.3	57.2	49.3	<0.001
IES cutoff (posttraumatic stress symptoms)	13.3	18.0	15.7	0.060
Worsened self-assessed mental health effect of SARS (very much or somehow)	42.6	54.1	48.4	0.001
Sleeping/psychosomatic problems				
Experienced trouble falling or staying asleep because of SARS (sometimes or often)	9.3	13.6	11.5	0.054
Sleep was restless in the last month (sometimes or often)	15.3	21.9	18.6	0.015
Experienced sweating, trouble breathing, nausea, or heart pounding because of SARS	5.2	8.5	6.9	0.059
Substance use				
Increased frequency of smoking‡	13.2	11.5	12.9	0.820
Increased frequency of drinking alcohol§	4.7	14.8	6.8	0.062
Perceived increased stress because of SARS				
Increased or much increased work stress	35.4	38.2	36.8	0.403
Increased or much increased family stress	38.6	37.0	37.8	0.639
Increased or much increased financial stress	25.1	28.0	26.5	0.344
Other problems				
Family members in need of psychology or psychiatry services	4.7	3.7	4.2	0.539
Difficult or very difficult to concentrate at work¶	18.8	21.8	20.1	0.409
Worsened or much worsened sexual life	6.2	5.9	6.1	0.855
Worsened or much worsened social life	31.0	43.4	37.2	<0.001
Family member with worsened or much worsened emotional states	26.0	26.9	26.5	0.783

*IES, Impact of event scale. †Chi-square test. ‡Among those who were smokers. §Among those who drank alcohol. ¶Among those who were currently working full time or part time.

Using the cutoff values of the IES of the combined intrusion and avoidance subscale (28), we observed that 13.3% of male respondents and 18.0% of female respondents (p = 0.060), respectively, had moderate or severe posttraumatic stress symptoms (1.3% and 1.5%, respectively, of the male and female respondents had severe symptoms) (Table 4). Female respondents had higher mental health quality of life (QOL) and vitality QOL subscale scores (p < 0.05).

A total of 36.8% and 37.8%, respectively, of the respondents perceived that the level of stress related to work and family had increased as a result of the SARS epidemic, and 26.5% of the respondents were facing increased financial stress. Among current smokers, 12.9% had increased their frequency of smoking during the SARS epidemic compared with the pre-SARS period. Among those who consumed alcohol, 4.7% of male respondents and 14.8% of female respondents had increased their frequency of drinking (Table 4).

Of the respondents, 11.5% had trouble falling or staying asleep because they had been preoccupied by thoughts related to SARS. In the month preceding the survey, 18.6% of the respondents reported that they slept restlessly (Table A1). A total of 6.9% of respondents had psychosomatic symptoms such as sweating, nausea, trouble breathing, or pounding heartbeats when thinking about the SARS epidemic (Table 4). When the situations before or during the SARS epidemic were compared, we observed that 4.2% of respondents had family members in need of psychological or psychiatric services, 6.1% reported poorer sexual functioning, 37.2% reported a poorer social life, 20.1% of those employed reported difficulty in concentrating on their work, and 26.5% of respondents reported poorer emotional states of their family members (Table 4).

### Factors Predicting Mental Health Effects

#### Stage 1 Analysis (Stepwise Regression of Sociodemographic Variables)

The relevant sociodemographic variables (Table 1) were entered as input variables in stepwise linear and logistic regression models to predict IES scores, mental health, and vitality QOL scores and various psychological effects (e.g., whether one had trouble falling asleep) (Tables A2 and A3).

#### Stage 2 Analysis (Adjusted for Variables Significant in Stage 1)

Those who felt horrified, apprehensive, and helpless because of SARS were more likely to report posttraumatic stress symptoms (as measured by IES) or have a lower mental health QOL and vitality QOL scores (Table A2). Those who felt apprehensive because of SARS were more likely to report sleeping problems and experience overall negative mental health effects ( Table A3). Feeling helpless because of SARS was associated with sleeping problems, while worrying about contracting SARS was associated with overall negative mental health and psychosomatic symptoms.

Increased work-related and family-related stress, but not increased financial stress, were associated with IES and mental health QOL and vitality QOL outcomes (Table A2). Increased work-related stress was also associated with sleeping problems, psychosomatic symptoms, and a poorer social life. Increased family-related stress was associated with a poorer social life, worsened mental health, and the need for psychological/psychiatric services (Table A3). Financial stress was associated with worsened sexual functioning and worsened mental health.

A poorer social life was associated with IES (intrusion and hyperarousal) (Table A2), sleeping problems, worsened sexual functioning, and a negative overall effect on mental health (Table A3). Worsened emotional states of family members was significantly associated with subscales of the IES (intrusion and avoidance) and QOL subscales, sleeping problems, worsened overall mental health effects, and worsened sexual and social life.

## Discussion

The general public in Hong Kong did not perceive the possibility of a resurgence of SARS. The degree of vigilance was high when respondents were asked about current preventive behaviors and hypothetical situations of having a few new SARS cases reported in Hong Kong. The entire city was expected to react strongly to a resurgence of SARS. However, some precautions may be unwarranted and could have a negative economic effect (29). Approximately 20% of respondents believed that they would be in a state of panic, 37% were still emotionally disturbed by SARS, and 4% had family members in need of psychological or psychiatric services. Thus, the general public needs to be better prepared psychologically to be able to avoid possible panic and emotional disturbances in a resurgence of SARS.

More than 90% of respondents perceived that mask use, frequent hand washing, and disinfection of living quarters are efficacious means of SARS prevention. Although the droplet theory of transmission has been widely accepted by the scientific community, other theories involving fomites (30), aerosols (5), sewage (31), rats (7), and wild animals (32) remain controversial. No conclusions have been reached regarding these topics. Information provided by health workers has also shown marked variations (33). In the absence of confirmed "top-down" official information, the general public has apparently been forming their own attitudes in a "bottom-up" manner. Similarly, another study claimed that laypersons in Hong Kong, Taiwan, and Toronto used "naive knowledge models" that were either incomplete or faulty in conceptualizing the symptoms, threat, spread, and prevention of SARS (34). Another study also reported substantial misinformation and false beliefs related to the existence of SARS in the general public (20). Therefore, it is important to understand how perceptions were formed during a newly emerging epidemic.

If one compares the results of this study with those obtained in March 2003, SARS-related perceptions and behaviors changed sharply over time (21,35). The results of several studies show that most of the general public had always believed that SARS could be transmitted through droplets, and increasingly more people believed that SARS is transmittable through fomites, but opinions about aerosol transmission of SARS remained split (20,21,35). Different studies had similar conclusions that perceptions such as perceived efficacy and perceived susceptibility were predictive of the use of preventive measures and emotional responses (20,21,35).

In survey 2, the prevalences of avoiding hospitals and China were 66.7% and 68.6%, respectively, which are comparable with the results obtained in another study conducted in May 2003 (21). More than 80% of respondents in this study would use a mask if they had influenza while traveling, while another study conducted from April 22 to April 29, 2003, documented that ≈70% would do so (36). A third study reported that ≈50% of the general public practiced at least 5 of 7 studied types of preventive measures (20). Preventive behaviors were thus prevalent throughout different phases of the epidemic.

A study conducted on approximately April 1, 2003 (20), reported that 12.6% of the respondents were quite or very anxious. Our survey 2, which was conducted at the ending phase of the epidemic, showed that ≈16% of the respondents had moderate or severe posttraumatic stress symptoms. Another study conducted from April 11 to May 19, 2003 (37), documented that ≈68% of healthy control participants experienced negative SARS-related effects. Our study showed that ≈48% assessed their mental health as being worse because of SARS. Also, 20% of the respondents worried about finances, whereas ≈27% of the respondents had financial stress. Emotional disturbance (our survey 2) and anxiety level (20) were associated with use of preventive measures. Psychological stress was prevalent throughout different phases of the epidemic.

Sex differences in perceptions and responses were observed. Men and women may have reacted differently to the incomplete evidence available when forming their views about the spread and control of SARS. Women were more likely than men to believe that SARS could be transmitted through different modes or that different methods could effectively prevent SARS.

A sizable proportion of the population felt horrified, apprehensive, or helpless because of the SARS epidemic in Hong Kong. Approximately 40%–50% of the respondents reported that their mental health status had been worsened, and 40% felt that their levels of work- and family-related stress had increased during the epidemic. The SARS epidemic exerted adverse effects on multiple aspects of social, family, sexual, and occupational domains. Those who smoked and drank in Hong Kong also increased their frequency of smoking and drinking. Thus, the mental health effect was prevalent and pervasive. Longitudinal studies are therefore required to understand the long-term mental health effects of SARS. Similar effects had been documented in studies conducted after the September 11, 2001, terrorist attack in the United States (15,38,39). Some similarities may exist in the community responses of different large-scale disasters.

Married persons tended to have a worsened mental health status because of SARS. Married people usually have a lower prevalence of psychological problems and a better support system compared with single people. However, ≈25% of respondents reported that their family members were emotionally affected by the epidemic, and ≈40% reported increased family stress. When an infectious epidemic is being faced, the worries of cross-infection and the well-being of family members are critical in determining the mental health effects of the epidemic on a person. Mental health services should take into account mutual influences among family members. Increased work-related stress was another predictor of mental health effects. Business activity decreased sharply, and the job security of many people was threatened. Similarly, social life was reported as worse among 40% of respondents. The effect of SARS was not confined to physical and psychological aspects, but it also affected socioeconomic and social aspects, which in turn determined the psychological well-being of persons.

This study had several limitations. First, data were self-reported and are subject to reporting biases. However, the interviews were anonymous. Second, some questions were asked about behavior in response to a potential resurgence of SARS, rather than measuring actual behavior because we were investigating how the general public would respond to a possible resurgence of SARS. Third, the response rates of the studies were moderate (≈58% in survey 1 and 65% in survey 2), and no data were available from nonresponders. The response rates were comparable with those of other survey studies in Hong Kong (40,41), and the age composition of the 2 samples was comparable with those of the Hong Kong census figures. Furthermore, we were not able to ascertain the previous psychological conditions of the respondents. However, results of the study should reflect the direct effect of SARS, rather than the general psychological status of the respondents. Some important factors, such as intensity of media exposure, were not measured in the study. However, many variables in this study (e.g., perceived reaction to resurgence and some psychological responses variables) have not been reported elsewhere.

SARS may return to some parts of the world, and preparative work is warranted. Up-to-date SARS-related knowledge should be collated and disseminated to the general public to promote effective public health measures and avoid unnecessary panic in case of a resurgence. Sex differences and concerns for family members and work need to be considered by relevant information campaigns. The perception of the general public changes rapidly over time and needs to be monitored closely. Bioterrorism may be similar to SARS in many ways. The results of this study predict that, in cases of bioterrorism, the general public would form their perceptions based on weak evidence, and the effect on mental health would also be evident. Modifying perceptions of the public would facilitate control of the disaster and alleviate panic among the general population. Further studies on the process of perception formation and its consequences on psychological responses in newly emerged epidemics are warranted.

This study was supported by the Chinese University of Hong Kong.

## Appendix

**Table A1:** Univariate and multivariate analyses of factors associated with preventive behaviors and emotional responses because of acute respiratory syndrome (SARS) (survey 1 data)*

	OR (95% CI)							
	Would avoid going to mainland China		Would avoid going to hospitals		Still emotionally disturbed because of SARS		Would be in a state of panic if there are sporadic SARS cases	
	Univariate	Multivariate†	Univariate	Multivariate†	Univariate	Multivariate†	Univariate	Multivariate†
Female sex (ref = male)	1.71 (1.25–2.34)	1.41 (1.01–1.97)	1.56 (1.16–2.11)	–	1.32 (1.00–1.75)	–	1.83 (1.29–2.61)	1.60 (1.08–2.26)
Age group 45–60 y (ref = 18–44 y)					1.84 (1.38–2.46)	–	1.52 (1.06–2.15)	–
Education level <12 y (ref = university)					1.82 (1.29–2.56)	1.48 (1.02–2.14)	–	–
Married/divorced/widowed (ref = single)	1.49 (1.08–2.06)	–	1.73 (1.27–2.35)	1.48 (1.06–2.07)	2.95 (2.12–4.12)	2.43 (1.71–3.44)	2.07 (1.37–3.13)	1.92 (1.23–2.97)
SARS is transmittable through respiratory droplets (ref = no)	1.76 (1.15–2.71)	–	–	–	1.54 (1.16–2.04)	–	2.08 (1.46–3.00)	1.75 (1.20–2.54)
SARS is transmittable through fomites (ref = no)	–		1.84 (1.21–2.79)	1.64 (1.06–2.56)	2.21 (1.56–3.14)	1.69 (1.16–2.45)	2.52 (1.54–4.10)	2.05 (1.23–3.41)
SARS is transmittable through aerosols (ref = no)	1.60 (1.17–2.19)	1.47 (1.05–2.05)	1.52 (1.13–2.06)	–	1.70 (1.26–2.30)	–	–	–
SARS is transmittable through rats and cockroaches (ref = no)	2.11 (1.51–2.95)	–	1.81 (1.31–2.52)	–	1.81 (1.11–2.97)	–	222 (1.09–2.05)	–
SARS is transmittable through pets (ref = no)	1.81 (1.33–2.49)	–	1.56 (1.15–2.11)	–	2.41 (1.59–3.67)	1.87 (1.20–2.90)	–	–
SARS is transmittable through sewage (ref = no)	2.19 (1.40–3.44)	–	–	–	1.61 (1.17–2.20)	1.41 (1.01–1.97)	–	–
High likelihood of contracting SARS through wild animal meat (ref = no)	3.51 (2.43–5.08)	2.71 (1.84–4.00)	2.86 (1.99–4.12)	–	1.35 (1.02–1.78)	–	–	–
Old people are more likely than others to contract SARS (ref = no)	–	–	1.86 (1.36–2.54)	1.55 (1.11–2.16)	1.40 (1.06–1.85)	–	–	–
There would be a resurgence of SARS in Hong Kong in the coming 6 months (ref = no)	2.33 (1.66–3.27)	–	2.03 (1.47–2.79)	1.55 (1.06–2.27)	1.69 (1.27–2.24)	1.62 (1.20–2.18)	1.73 (1.22–2.44)	1.48 (1.03–2.13)
There would be a resurgence of SARS in China in the coming 6 months (ref = no)	2.71 (1.96–3.73)	2.40 (1.71–3.35)	2.01 (1.47–2.75)	1.50 (1.03–2.18)	–	–	–	–
There would be a resurgence of SARS overseas in the coming 6 months (ref = no)	1.63 (1.14–2.34)	–	–	–	–	–	0.52 (0.38–0.77)	0.56 (0.38–0.82)
The government could control SARS if there were sporadic new SARS cases in Hong Kong (ref = no)	–	–	–	–	–	–	0.50 (0.34–0.74)	0.52 (0.34–0.79)
I am still emotionally disturbed because of SARS (ref = no)	2.44 (1.71–3.49)	2.04 (1.41–2.97)	2.08 (1.49–2.90)	1.63 (1.14–2.33)	–	–	4.99 (3.45–7.22)	–

*OR, odds ratio; CI, confidence interval; ref, referent; –, non-significant in multivariate analysis (although significant in univariate analysis). †Multivariate odds ratio (stepwise logistic regression) using univariate significant variables as input variables. Belief that SARS is transmittable through droplets or fomites, belief that there is no effective drugs to treat SARS, and belief that there would be a resurgence of SARS in China in the coming 6 months were not univariately significant for any of the 4 dependent variables (data not tabulated).

**Table A2:** Factors predicting IES and QOL scores (stepwise linear regression models) (survey 2 data)*

	β coefficient (SE)				
	DV = IES subscale scores			DV = QOL (SF-36) subscale scores	
	Avoidance	Intrusion	Hyperarousal	Mental health	Vitality
Stage 1 (Stepwise selection of significant sociodemographic factors)					
Female sex (ref = male)	NS	1.62† (0.48)	1.07† (0.25)	NS	–4.66† (1.47)
Age group, y (ref = 18–24)					
25–34	NS	NS	NS	NS	NS
35–49	NS	NS	NS	NS	NS
>50	NS	NS	NS	NS	NS
Education level 10–11 y (ref = <9 y)	NS	NS	NS	NS	NS
Pre-university	–1.27† (0.45)	NS	NS	NS	NS
University or higher	NS	NS	NS	NS	NS
Marital status (ref = single)					
Currently married or lived together	NS	1.34† (0.49)	0.72† (0.26)	NS	NS
Divorced or widow	NS	NS	NS	NS	–12.73‡ (5.63)
Employment status (ref = full time)					
Part time	NS	NS	NS	NS	NS
Unemployed	NS	NS	NS	NS	NS
Housewife	NS	NS	NS	–6.03† (1.82)	NS
Student	NS	NS	NS	5.97† (2.17)	4.81‡ (2.31)
Retired or other	NS	NS	NS	NS	NS
Religion (ref = no)					
Christian	NS	NS	NS	NS	NS
Buddhist	NS	2.98† (0.88)	2.43† (0.46)	–6.56† (2.51)	NS
Other	NS	NS	NS	NS	NS
Stage 2 (Stepwise selection of psychological variables adjusting for univariately significant sociodemographic factors)					
Feel horrified because of SARS (ref = moderately to strongly disagree)					
Agree or strongly agree	–	–	0.87‡ (0.30)	–	–
Feel apprehensive because of SARS (ref = moderately to strongly disagree)					
Agree or strongly agree	1.13† (0.42)	2.47† (0.48)	0.97† (0.28)	–8.52† (1.37)	–5.68† (1.58)
Felt helpless about SARS (ref = moderately to strongly disagree)					
Agree or strongly agree	1.12† (0.40)	–	–	–2.82‡ (1.28)	–3.78‡ (1.47)
Worried about oneself or family member contracting SARS (ref = moderately worried to not worried)					
Worried or very much worried	1.47† (0.40)	2.35† (0.45)	1.31† (0.24)	–4.51† (1.27)	–3.45‡ (1.48)
Increased work stress because of SARS (ref = same to much decreased)					
Increased or much increased	NS	1.58† (0.47)	0.74‡ (0.25)	–4.96† (1.35)	NS
Increased financial stress because of SARS (ref = same to much decreased)					
Increased or much increased	–	–	–	–	–3.15‡ (1.55)
Increased family stress because of SARS (ref = same to much decreased)					
Increased or much increased	1.28† (0.46)	1.09‡ (0.53)	0.83‡ (0.27)	–5.69† (1.51)	–3.96‡ (1.77)
Change in social life because of SARS (ref = same to much improved)					
Worse or much worse	–	1.27‡ (0.50)	1.15† (0.25)	–	–
Change in family members' emotional states because of SARS (ref = same to much improved)					
Worse or much worse	1.16‡ (0.46)	2.21† (0.55)	–	–6.20† (1.48)	–4.48† (1.70)
Adjusted R^2^	0.081	0.226	0.243	0.227	0.106
Range of scores	0–35	0–35	0–20	6–30	4–24

*IES, impact of event scale; QOL, quality of life; DV, dependent variable; SF36, Medical Outcomes Study 36-Item Short Form Health Survey; SE, standard error; ref, referent; NS, not selected in Stage 1 stepwise linear regression analysis; –, not selected in Stage 2 stepwise linear regression analysis; SARS, severe acute respiratory syndrome. Perceived severity of SARS was entered into the stage 2 analysis, but it was not selected by any of the models. †p < 0.01. ‡p < 0.05.

**Table A3:** Factors associated with other psychological variables (stepwise logistic regression model) (survey 2 data)*

	OR (95% CI)					
	Having trouble falling/ staying asleep (yes = 1)	Having a perceived worsened sexual life (yes = 1)	Having a psychosomatic response (yes = 1)	Having a perceived worsened social life (yes = 1)	Having a perceived need for a psychiatrist or psychologist (yes = 1)	Having a perceived overall effect on mental health (yes = 1)
Stage 1 (Stepwise selection of sociodemographic factors)						
Female (ref = male)	NS	NS	NS	1.59† (1.19–2.14)	NS	1.62† (1.22–2.15)
Age group, y (ref = 18–24)						
25–34	NS	9.96‡ (1.26–94.50)	NS	2.02† (1.25–3.28)	NS	1.64‡ (1.07–2.53)
35–49	NS	12.58‡ (1.67–94.50)	NS	2.78† (1.80–4.29)	NS	NS
>50	NS	18.26† (2.35–141.68)	NS	2.32† (1.41–3.82)	NS	1.87† (1.19–2.95)
Education level (ref = 9 y)						
10–11 y	NS	NS	NS	NS	NS	NS
Pre-university	NS	NS	NS	NS	NS	NS
University or higher	0.42† (0.23–0.76)	NS	NS	NS	NS.	NS
Marital status (ref = single)						
Currently married/cohabited	NS	NS	NS	NS	NS	NS
Divorced or widow	5.38† (1.63–17.73)	NS	NS	NS	NS	NS
Employment status (ref = full time)						
Part time	NS	NS	NS	NS	NS	NS
Unemployed	2.98† (1.60–5.56)	NS	NS	NS	NS	NS
Housewife	2.28† (1.59–4.14)	NS	2.77† (1.43–5.39)	NS	NS	NS
Student	NS	NS	NS	NS	NS	NS
Retired or other	NS	NS	NS	NS	NS	NS
Religion (ref = no religion)						
Christian	NS	NS	NS	NS	NS	NS
Buddhist	NS	2.91† (1.31–6.46)	2.57‡ (1.21–5.46)	NS	NS	NS
Other	NS	NS	NS	NS	NS	NS
Stage 2 (Stepwise selection of psychological variables adjusting for univariately significant sociodemographic factors by using "enter" syntax )						
Perceived severity of SARS (ref = moderate to not severe)						
Severe or very severe	–	–	–	–	–	2.20† (1.11–4.35)
Feel apprehensive because of SARS (ref = moderately to strongly disagree)						
Agree or strongly agree	2.32† (1.38–3.89)	–	–	–	–	1.90† (1.35–2.68)
Felt helpless because of SARS (ref = moderately to strongly disagree)						
Agree or strongly agree	1.75‡ (1.11–2.75)	–	–	–	–	–
Worried about oneself or family member contracting SARS (ref = moderately worried to not worried)						
Worried or very much worried	–	–	2.25† (1.22–4.14)	–	–	2.10† (1.53–2.89)
Increased work stress because of SARS (ref = same to much decreased)						
Increased or much increased	2.67† (1.07–2.62)	–	2.57† (1.41–4.70)	1.92† (1.36–2.70)	–	–
Increased family stress because of SARS (ref = same to much decreased)						
Increased or much increased	–	–	–	1.50‡ (1.03–2.18)	2.39‡ (1.17–4.90)	1.56‡ (1.06–2.30)
Increased financial stress because of SARS (ref = same to much decreased)						
Increased or much increased	–	1.96‡ (1.05–3.67)	–	–	–	1.45‡ (1.04, 2.04)
Change in social life because of SARS (ref = same to much improved)						
Worse or worse	2.24† (1.43–3.49)	2.24‡ (1.12–4.48)	–	NA	–	1.78† (1.26–2.53)
Change in family members' emotional states (ref = same to much improved)						
Worse or much worse	1.68‡ (1.07–2.62)	2.78‡ (1.43–5.37)	–	5.56† (3.84–8.05)	–	2.25† (1.51–3.35)

*OR, odds ratio; CI, confidence interval; ref, referent; NS, not selected in stage 1 stepwise logistic regression analysis; SARS, severe acute respiratory syndrome; –, not selected in stage 2 stepwise logistic regression analysis; NA, not applicable. The variable "feel horrified because of SARS" was considered by the stage 2 stepwise analysis, but was not selected by any of the 6 models. †p < 0.01. ‡p < 0.05.

## References

[1] World Health Organization. Summary table of SARS cases by country, November 1 2002–August 7, 2003. [cited 2003 Sep 15]. Available from http://www.who.int/csr/sars/country/2003_08_15/en/

[2] World Health Organization. World Health Organization changes Hong Kong, Guangdong travel recommendations. [cited 2003 Aug 5]. Available from http://www.who.int/mediacentre/releases/2003/prwha4/en/

[3] Education and Manpower Bureau of Hong Kong. Suspension of classes for the prevention of atypical pneumonia. [cited 2003 Aug 5]. Available from http://www.info.gov.hk/gia/general/200303/27/0327269.htm

[4] Sim K, Chua HC. The psychological impact of SARS: a matter of heart and mind. CMAJ. 2004;170:811–2. 10.1503/cmaj.1032003 14993176 PMC343855

[5] Yu IT, Li Y, Wong TW, Tam W, Chan AT, Lee JH, Evidence of airborne transmission of the severe acute respiratory syndrome virus. N Engl J Med. 2004;350:1731–9. 10.1056/NEJMoa032867 15102999

[6] Xu HF, Wang M, Zhang ZB, Zou XZ, Gao Y, Liu XN, An epidemiologic investigation on infection with severe acute respiratory syndrome coronavirus in wild animals traders in Guangzhou. Zhonghua Yu Fang Yi Xue Za Zhi. 2004;38:81–3. 15061910

[7] Ng SK. Possible role of an animal vector in the SARS outbreak at Amoy Gardens. Lancet. 2003;362:570–2. 10.1016/S0140-6736(03)14121-9 12932393 PMC7134840

[8] World Health Organization. Severe acute respiratory syndrome (SARS) in Taiwan, China. [cited 2004 May 19]. Available from http://www.who.int/csr/don/2003_12_17/en/

[9] World Health Organization. China confirms SARS infection in another previously reported case; summary of cases to date - Update 5. [cited 2004 May 10]. Available from http://www.who.int/csr/don/2004_04_30/en/

[10] World Health Organization. New case of laboratory-confirmed SARS in Guangdong, China - update 5. [cited 2004 Feb 3]. Available from http://www.who.int/csr/don/2004_01_31/en/

[11] Lau JTF, Tsui H, Lau M, Yang X. SARS transmission, risk factors, and prevention in Hong Kong. Emerg Infect Dis. 2004;10:587–92. 15200846 10.3201/eid1004.030628 PMC3323085

[12] Wu J, Xu F, Zhou W, Feikin D, Lin C-Y, He X, Risk factors for SARS among persons without known contact with SARS patients, Beijing, China. Emerg Infect Dis. 2004;10:210–6. 15030685 10.3201/eid1002.030730 PMC3322931

[13] Ahern J, Galea S, Resnick H, Kilpatrick D, Bucuvalas M, Gold J, Television images and psychological symptoms after the September 11, 2001, terrorist attacks. Psychiatry. 2002;65:289–300. 10.1521/psyc.65.4.289.20240 12530330

[14] Cardenas J, Williams K, Wilson JP, Fanouraki G, Singh A. PSTD, major depressive symptoms, and substance abuse following September 11, 2001, in a midwestern university population. Int J Emerg Ment Health. 2003;5:15–28. 12722486

[15] Vlahov D, Galea S, Resnick H, Ahern J, Boscarino JA, Bucuvalas M, Increased use of cigarettes, alcohol, and marijuana among Manhattan, New York, residents after the September 11th terrorist attacks. Am J Epidemiol. 2002;155:988–96. 10.1093/aje/155.11.988 12034577

[16] Boscarino JA, Galea S, Ahern J, Resnick H, Vlahov D. Utilization of mental health services following the September 11th terrorist attacks in Manhattan, New York City. Int J Emerg Ment Health. 2002;4:143–55. 12387188

[17] Wolinsky FD, Wyrwich KW, Kroenke K, Babu AN, Tierney WM. 9-11, personal stress, mental health, and sense of control among older adults. J Gerontol B Psychol Sci Soc Sci. 2003;58:S146–50. 10.1093/geronb/58.3.S146 12730315

[18] Kawana N, Ishimatsu S, Kanda K. Psycho-physiological effects of the terrorist sarin attack on the Tokyo subway system. Mil Med. 2001;166(Suppl):23–6. 11778423

[19] Bleich A, Gelkopf M, Solomon Z. Exposure to terrorism, stress-related mental health symptoms, and coping behaviors among a nationally representative sample in Israel. JAMA. 2003;290:612–20. 10.1001/jama.290.5.612 12902364

[20] Leung GM, Lam TH, Ho LM, Ho SY, Chan BH, Wong IO, The impact of community psychological responses on outbreak control for severe acute respiratory syndrome in Hong Kong. J Epidemiol Community Health. 2003;57:857–63. 10.1136/jech.57.11.857 14600110 PMC1732323

[21] Lau JT, Yang X, Tsui H, Kim JH. Monitoring community responses to the SARS epidemic in Hong Kong: from day 10 to day 62. J Epidemiol Community Health. 2003;57:864–70. 10.1136/jech.57.11.864 14600111 PMC1732318

[22] Department of Census and Statistics. Population by ethnicity, 2001. Main tables of the 2001 population. [cited 2003 Aug 21]. Available from Census http://www.info.gov.hk/censtatd/eng/hkstat/fas/01c/cd0052001_index.html

[23] Horowitz M, Wilner M, Alvarez W. Impact of event scale: a measure of subjective stress. Psychosom Med. 1979;41:209–18. 472086 10.1097/00006842-197905000-00004

[24] Weiss D, Marmar C. The impact of event scale. In: Wilson J, Keane T, editors. Assessing psychological trauma and PTSD. New York: Guildford Press; 1997.

[25] Ware JE Jr, Sherbourne CD. The MOS 36-item short-form health survey (SF-36). I. Conceptual framework and item selection. Med Care. 1992;30:473–83. 10.1097/00005650-199206000-00002 1593914

[26] Lam CL, Gandek B, Ren XS, Chan MS. Tests of scaling assumptions and construct validity of the Chinese (HK) version of the SF-36 Health Survey. J Clin Epidemiol. 1998;51:1139–47. 10.1016/S0895-4356(98)00105-X 9817131

[27] Lam CL, Lauder IJ, Lam TP, Gandek B. Population based norming of the Chinese (HK) version of the SF-36 health survey. Hong Kong Practitioner. 1999;21:460–70.

[28] Devilly GJ. Assessment devices. The University of Melbourne, Forensic Psychology and Victim Services. [cited 2003 Aug 11]. Available from http://www.criminology.unimelb.edu.au/victims/resources/assessment/assessment.html

[29] Blendon RJ, Benson JM, DesRoches CM, Raleigh E, Taylor-Clark K. The public's response to severe acute respiratory syndrome in Toronto and the United States. Clin Infect Dis. 2004;38:925–31. 10.1086/382355 15034821

[30] Lau JTF, Fung KS, Wong TW, Kim JH, Wong E, Chung S, SARS transmission among hospital workers in Hong Kong. Emerg Infect Dis. 2004;10:280–6. 15030698 10.3201/eid1002.030534 PMC3322933

[31] Inadequate plumbing systems probably contributed to SARS transmission. Wkly Epidemiol Rec. 2003;78:371–2. 14587210

[32] Guan Y, Zheng BJ, He YQ, Liu XL, Zhuang ZX, Cheung CL, Isolation and characterization of viruses related to the SARS coronavirus from animals in southern China. Science. 2003;302:276–8. 10.1126/science.1087139 12958366

[33] Thompson DR, Lopez V, Lee D, Twinn S. SARS - a perspective from a school of nursing in Hong Kong. J Clin Nurs. 2004;13:131–5. 10.1046/j.1365-2702.2003.00884.x 14723663 PMC7185828

[34] Slaughter LA, Patel VL. A study of laypersons' mental models and information needs concerning severe acute respiratory syndrome (SARS). Medinfo. 2004;2004:1866.

[35] Tang CS, Wong CY. An outbreak of the severe acute respiratory syndrome: predictors of health behaviors and effect of community prevention measures in Hong Kong, China. Am J Public Health. 2003;93:1887–8. 10.2105/AJPH.93.11.1887 14600058 PMC1448068

[36] Lau JT, Yang X, Tsui H, Pang E, Kim JH. SARS preventive and risk behaviours of Hong Kong air travellers. Epidemiol Infect. 1999;132:727–36. 10.1017/S0950268804002225 15310175 PMC2870154

[37] Chua SE, Cheung V, Cheung C, McAlonan GM, Wong JW, Cheung EP, Psychological effects of the SARS outbreak in Hong Kong on high-risk health care workers. Can J Psychiatry. 2004;49:391–3. 15283534 10.1177/070674370404900609

[38] Galea S, Ahern J, Resnick H, Kilpatrick D, Bucuvalas M, Gold J, Psychological sequelae of the September 11 terrorist attacks in New York City. N Engl J Med. 2002;346:982–7. 10.1056/NEJMsa013404 11919308

[39] Silver RC, Holman EA, McIntosh DN, Poulin M, Gil-Rivas V. Nationwide longitudinal study of psychological responses to September 11. JAMA. 2002;288:1235–44. 10.1001/jama.288.10.1235 12215130

[40] Lau JT, Tang AS, Tsui HY. The relationship between condom use, sexually transmitted diseases, and location of commercial sex transaction among male Hong Kong clients. AIDS. 2003;17:105–12. 10.1097/00002030-200301030-00014 12478075

[41] Lau JT, Tsui HY. Surveillance of HIV/AIDS-related attitudes and perceptions among the general public in Hong Kong from 1994 to 2000. AIDS Educ Prev. 2002;14:419–31. 10.1521/aeap.14.6.419.24080 12413187

